# ProMED-mail: background and purpose.

**DOI:** 10.3201/eid0707.017736

**Published:** 2001

**Authors:** J Woodall, C H Calisher

**Affiliations:** ProMED-mail, Rio de Janeiro, Brazil. woodall@bioqmed.ufrj.br


### ProMED-mail: Background and Purpose

**Jack Woodall* and Charles H. Calisher†**
*ProMED-mail, Rio de Janeiro, Brazil and †Colorado State University, Fort Collins, Colorado, USA

The online program to monitor emerging diseases (ProMED-mail) was established in 1994 with the support and encouragement of the Federation of American Scientists and SatelLife. The principal intent of ProMED-mail is to assist local, national, and international organizations in disseminating, as rapidly as possible, reports of outbreaks of emerging infectious diseases wherever they occur; these reports are taken from sources such as media reports, online summaries, local observers, official reports, and others. Subscribers are encouraged to contribute reports and to participate in the dialogue.

With a minimum amount of funding, volunteer moderators, and a bulk mailer in Newfoundland, ProMED-mail grew from 40 subscribers in 1994 to its present number of over 20,000 in more than 160 countries; there are no subscription fees. Since 1999, ProMED-mail has been administered by the International Society for Infectious Diseases, with servers and software furnished by Oracle, Inc. The e-mail service provider is located in the Harvard School of Public Health.

At a "Meet the Professor" session of the 2000 International Conference on Emerging Infectious Diseases," ProMED-mail staff described the history and purpose of the list-serve and fielded a wide range of queries from an attentive and supportive audience, most of whom were already familiar with ProMED-mail but not, it turned out, with the website <www.promedmail.org>.

"Would ProMED-mail report the occurrence of Ebola disease if it occurred in New York City?" Of course. "Has ProMED-mail felt constrained by governments or individuals in governments from reporting disease occurrences?" No. "Would ProMED-mail report a very small cluster of cases?" It depends on the etiology. "Does ProMED-mail report rumors?" No, but when we hear a rumor we might post a Request for Information. "How many hours per day, on average, do moderators spend working on ProMED-mail?" Three to six hours, depending on their area of responsibility. "Does ProMED-mail consider the World Health Organization a competitor?" Absolutely not. WHO, Office International des Epizooties, and other international and national organizations are able to report only what is officially reported to them. ProMED-mail has no such constraints, thus it is able to post preliminary and unofficial reports and summaries.

It is clear from these and other questions and comments and from the number of subscribers, many of whom are from national and international health agencies, that ProMED-mail fills a void. It is also clear that other, more official reporting systems are needed. These should follow the example of the outbreak page on WHO's website <http://www.who.int/disease-outbreak-news/index.html>, which gives short summaries of outbreaks as they are received. It would also be encouraging to see individual countries adapting the independent ProMED-mail format to supplement their own national outbreak reporting systems.

**Acknowledgments**

The writers wish to acknowledge the other members of the ProMED-mail team: Daniel S. Shapiro (managing editor), Edward W. Schroder, Timothy Brewer, Peter Cowen, Luiz Jacintho da Silva, Tam Garland, Richard I. Hamilton, Martin Hugh-Jones, Susan M. Krumplitsch, Stephen S. Morse, Jonathan Nash, Eskild Peterson, Marjorie P. Pollack, Craig R. Pringle, Alan Ronald, Barbara Hatch Rosenberg, Norman Stein, Phil Temples, and Alexander Vladyko.

Address for correspondence: Jack Woodall, Depto. de Bioquimica Medica, Instituto de Ciencias Biomedicas, Universidade Federal do Rio de Janeiro, Cidade Universitaria, Rio de Janeiro-RJ-21941-590, Brazil; fax: 5521-270-8647; e-mail: woodall@bioqmed.ufrj.br

